# Revealing the Neuroimaging Mechanism of Acupuncture for Poststroke Aphasia: A Systematic Review

**DOI:** 10.1155/2022/5635596

**Published:** 2022-04-21

**Authors:** Boxuan Li, Shizhe Deng, Bomo Sang, Weiming Zhu, Bifang Zhuo, Menglong Zhang, Chenyang Qin, Yuanhao Lyu, Yuzheng Du, Zhihong Meng

**Affiliations:** ^1^First Teaching Hospital of Tianjin University of Traditional Chinese Medicine, Tianjin, China; ^2^National Clinical Research Center for Chinese Medicine Acupuncture and Moxibustion, Tianjin, China; ^3^Tianjin University of Traditional Chinese Medicine, Tianjin, China

## Abstract

**Background:**

Aphasia is a common symptom in stroke patients, presenting with the impairment of spontaneous speech, repetition, naming, auditory comprehension, reading, and writing function. Multiple rehabilitation methods have been suggested for the recovery of poststroke aphasia, including medication treatment, behavioral therapy, and stimulation approach. Acupuncture has been proven to have a beneficial effect on improving speech functions in repetition, oral speech, reading, comprehension, and writing ability. Neuroimaging technology provides a visualized way to explore cerebral neural activity, which helps reveal the therapeutic effect of acupuncture therapy. In this systematic review, we aim to reveal and summarize the neuroimaging mechanism of acupuncture therapy on poststroke aphasia to provide the foundation for further study.

**Methods:**

Seven electronic databases were searched including PubMed, Web of Science, Embase, Cochrane Central Register of Controlled Trials, China National Knowledge Infrastructure, the Wanfang databases, and the Chinese Scientific Journal Database. After screening the studies according to the inclusion and exclusion criteria, we summarized the neuroimaging mechanism of acupuncture on poststroke aphasia, as well as the utilization of acupuncture therapy and the methodological characteristics.

**Result:**

After searching, 885 articles were retrieved. After removing the literature studies, animal studies, and case reports, 16 studies were included in the final analysis. For the acupuncture type, 10 studies used manual acupuncture and 5 studies used electroacupuncture, while body acupuncture (10 studies), scalp acupuncture (7 studies), and tongue acupuncture (8 studies) were applied for poststroke aphasia patients. Based on blood oxygen level-dependent (BOLD) and diffusion tensor imaging (DTI) technologies, 4 neuroimaging analysis methods were used including amplitude of low-frequency fluctuation (ALFF), regional homogeneity (ReHo), seed-based analysis, and independent component analysis (ICA). Two studies reported the instant acupuncture effect, and 14 studies reported the constant acupuncture's effect on poststroke aphasia patients. 5 studies analyzed the correlation between the neuroimaging outcomes and the clinical language scales.

**Conclusion:**

In this systematic review, we found that the mechanism of acupuncture's effect might be associated with the activation and functional connectivity of language-related brain areas, such as brain areas around Broca's area and Wernicke's area in the left inferior temporal gyrus, supramarginal gyrus, middle frontal gyrus, and inferior frontal gyrus. However, these studies were still in the preliminary stage. Multicenter randomized controlled trials (RCT) with large sample sizes were needed to verify current evidence, as well as to explore deeply the neuroimaging mechanisms of acupuncture's effects.

## 1. Introduction

Aphasia is an acquired language malfunction caused by the disorder of the speech center [1]. Stroke is the top leading cause of disability in China and is the major cause of mortality [2]. Each year, approximately 2 million Chinese people get attacked by the new-onset stroke [3]. Aphasia is a grave symptom of stroke, presenting with language malfunctions including spontaneous speech, repetition, naming, auditory comprehension, reading, and writing [4]. According to epidemiology research, the incidence of aphasia after the first stroke onset ranges from 23% to 38% [5–7]. Compared with the nonaphasia poststroke patients, the patient's mortality rate in the hospital is nearly 2 times higher and the hospital stay is 1.6 times longer [8, 9]. Chronic aphasia devastates patients' social participation and life quality. Moreover, the costs of aphasia rehabilitation are considerable, which aggravates the health economic burden [10]. It is estimated that the expense of rehabilitation varies from $89 to $864 according to the severity of aphasia [11], and prolonged hospital stay (0.66 days) contributes to high hospitalization costs ($971.35) in poststroke aphasia patients [12].

Multiple approaches have been used for aphasia treatment such as medication treatment, behavioral therapy, and stimulation approach [4, 13, 14]. Acupuncture originates from Traditional Chinese Medicine, and it has been practiced to treat aphasia for more than 3000 years. Plenty of studies have proven the therapeutic effect of acupuncture on aphasia, and a lot of evidence has indicated the beneficial effect of acupuncture on improving speech functions, including repetition, oral speech, reading, comprehension, and writing [15, 16]. Alone with speech and language therapies, acupuncture has become a pervasive treatment for poststroke aphasia [17]. Among the various acupuncture therapies, scalp acupuncture and tongue acupuncture are indispensable for poststroke aphasia. Both of their theories were developed based on the combination of the traditional Chinese meridian theory and the holographic theory [18, 19]. It has been demonstrated that scalp acupuncture has a beneficial effect on improving the daily activity ability of stroke patients [20]. Meanwhile, neural imaging technology provides a visualized way to reveal the brain activity mechanism of scalp acupuncture [21, 22]. Recent studies reported the effectiveness pathways of acupuncture on language function [23–25]. However, the mechanism of the recovery process is not fully illustrated. Clinical researches showed multiform language function impairments and the aphasia recovery process [26]. Apart from the relationship based on the structure-function hypothesis, evidence has been found in the variation of cerebral blood perfusion, brain functional connection, and neural activity, which enriched the mechanism of aphasia recovery [27–29]. Taking advantage of multidisciplinary combinations such as neuroimaging technology, researchers can explore the aphasia recovery mechanism in detail.

The functional magnetic resonance imaging (fMRI) provides a noninvasive way to explore the brain neural activity, which is a helpful tool to reveal the therapeutic effect of both instant acupuncture and constant acupuncture therapies [30]. The resting-state fMRI (rsfMRI) is proposed to be a practical approach to investigate spontaneous brain activity, which helps uncover the pathological mechanism. Accordingly, task-based fMRI is viewed as a tool to reflect the task-response properties evoked by specific stimulates [31]. Multiple analysis approaches have been applied to display the characteristics of the rsfMRI signals. ALFF is suggested to measuring the amplitude of fluctuation in the low-frequency range of neural activity in the resting state directly [32, 33]. It offers evidence of spontaneous brain activity at a single voxel level. In previous researches, ALFF is applied to study the effect of acupuncture therapy [34, 35]. ReHo is considered a useful tool to detect the regional functional homogeneity of neural synchronization, which provides features of brain connection within a specified voxel and the neighboring voxels [36]. Degree centrality is viewed to characterize the functional connectivity in the information communication of the brain network. It presents the single voxel value according to the functional connectivity strength within the brain network [37]. Functional connectivity (FC) is utilized to reveal the functional information communication between brain regions that are separated at the anatomic level, and ICA is proposed to explore the independent spatial sources of neural activity based on the whole brain function [38, 39].

With the use of neuroimaging technology, researchers make efforts to illustrate the central pathway of acupuncture's effect on poststroke aphasia. Acupuncture was taken as stimulation, and its instant effect was detected in the task-based fMRI [40]. Previously, researchers found the brain activation evoked by electroacupuncture was remarkably consistent with the activation induced by the image naming task, which included the left inferior frontal gyrus that accounts for the speech function [41]. Accordingly, rsfMRI was used to evaluate the cerebral functional state before and after acupuncture treatment. Based on the classical language function theory, studies focused on the anatomic structural regions proved that the decreased ALFF value in the left temporal pole was correlated with the naming function [42]. Recent studies revealed the connectivity in the surviving brain regions, which played a role in brain network studies [43, 44]. It has been verified that acupuncture could strengthen the language-related brain network in the left hemisphere [45].

The classic hypothesis of aphasia focused on the cortical lesions such as Broca's area and Wernicke's area. Using the MRI technology, researchers uncovered the damage of subcortical grey matter by scanning the two brains of Paul Broca's patients [46], while recent studies reported the new findings that neither the long-term aphasia condition nor the damage of anterior arcuate fasciculus can be fully explained by the infarction of Broca's area [47]. Other studies devoted to the brain network mechanism found disparate pathways in the aphasia recovery process [48]. Yet, the relationship between clinical effects and neuroimaging changes remains unclear. Hence, the objective of this review is to analyze the possible relationship between acupuncture stimulation, clinical effects, and cerebral response. Meanwhile, using the systematic review method, we aim to summarize the current acupuncture method, neuroimaging techniques, and potential cerebral mechanism of poststroke aphasia recovery.

## 2. Method

### 2.1. Included Criteria

- *Types of studies*: published randomized and nonrandomized controlled clinical studies of acupuncture on poststroke aphasia in English and Chinese
- *Participants*: patients that were diagnosed with aphasia following a stroke in WHO criteria; the type of aphasia was not limited.
- *Interventions*: acupuncture therapy included manual acupuncture (MA), electroacupuncture (EA), scalp acupuncture, and tongue acupuncture or combined with the control group treatment

### 2.2. Searching Strategies

We searched 7 electronic databases including PubMed, Web of Science, Embase, Cochrane Central Register of Controlled Trials, China National Knowledge Infrastructure, the Wanfang databases, and the Chinese Scientific Journal Database from January 2009 to September 2021 for relevant studies. References lists of identified publications, conference literature, and bibliographies of reviews were also inspected for further literature. The searching strategy and searching terms are listed in Table 1. The Preferred Reporting Items for Systematic Reviews and Meta-Analyses (PRISMA) statement was followed, and the PRISMA 2020 Checklist was attached in the supplemental material (available here) [49].

### 2.3. Study Selection

After searching, 885 articles were put into NoteExpress software (version 3.2.0). After removing the duplicated studies, the remaining 870 articles were screened by two reviewers through browsing titles and abstracts. Then, 68 remaining articles were selected by reading the full texts according to the inclusion criteria and exclusion criteria. Eventually, 16 articles got included in the final analysis. Disagreements were solved by consulting the third reviewer.

### 2.4. Data Extraction

The data extraction table was preset by BXL using Excel software, following the PRISMA statement and the STandards2 for Reporting Interventions in Controlled Trails of Acupuncture (STRICTA) guideline. The following information from the eligible studies was recorded by BMS and WMZ: publishing year, author, funding organization, study type, sample size, participants' information (stroke type, aphasia duration, and handedness), intervention details (needle session, needle duration, needle frequency, needle type, acupoint, needle response, control interventions, and treatment duration), and outcome details (outcome index, neuroimaging technologies, scanning design, image acquisition time, and neuroimaging results).

### 2.5. Data Analysis

In this systematic review, the characteristics of the included studies were analyzed using the bibliometric method. Then, the risk of bias assessment was conducted using the *Cochrane risk-of-bias tool for randomized trials* (*RoB 2*) [50, 51]. Finally, the neuroimaging outcomes were summarized to provide neural mechanism evidence of acupuncture for constant effect and instant effect on poststroke aphasia patients.

## 3. Result

### 3.1. Study Overview

**Figure 1:**
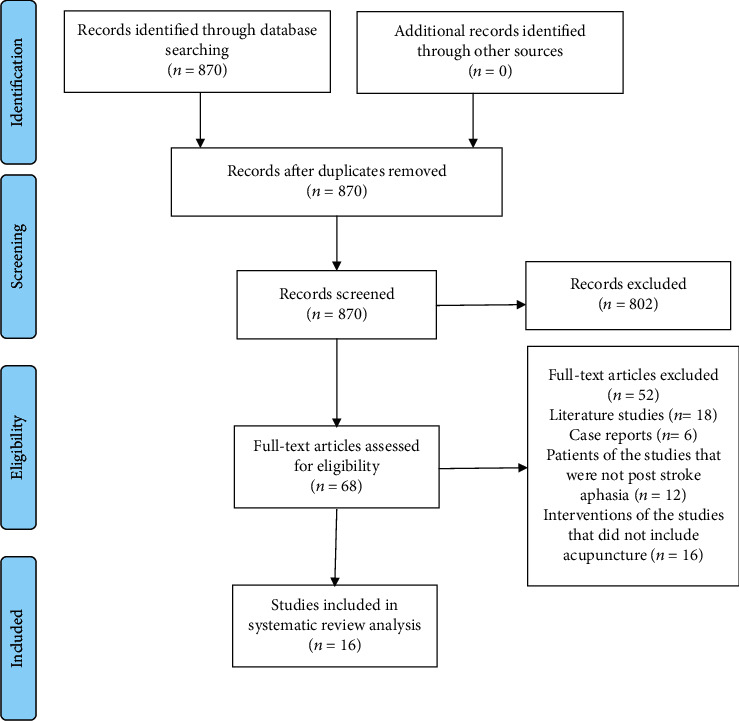
The flow diagram of literature screening.

**Figure 2:**
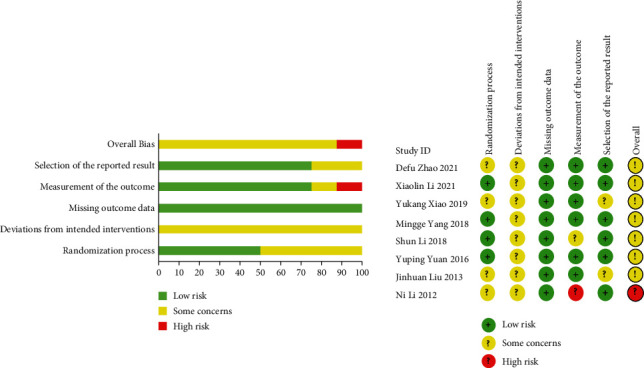
Risk of bias assessment of included randomized controlled trials.

A total of 885 studies were searched. After removing the duplicated studies, reviews, animal studies, and other ineligible clinical studies according to the screening protocol, 16 studies remained for the final analysis (Figure 1). The publishing date of the included studies was from March 2010 to August 2021. All of the 16 studies were conducted in China. The included 16 studies listed the funding organizations or the ethical committee. For the study type, there were 8 RCTs with 390 patients, 8 observational studies with 156 patients, and 96 healthy volunteers. The sample size ranges from 7 to 100. One study performed the blinding procedure on patients and used the sealed envelope to conduct the allocation concealment. The risk of bias assessment of included randomized controlled trials is summarized in Figure 2. When evaluating the randomization process, we took matched factors including gender, age, level of education, and disease duration (poststroke aphasia) into account. 4 studies that did not completely match these factors were graded as having some concerns. As for the deviation from intended intervention, the 8 included RCTs did not perform a blinding procedure for acupuncturists, and only one study reported conducting blinding procedures for participants. As a result, the included 8 RCTs were graded as having some concerns. All the 8 included RCTs reported the data in detail, while one study which did not describe the assessment procedure was graded as having some concerns; another study did not report the blinding of outcome assessor and was graded as high risk. For the part on the selection of the reported results, two studies did not illustrate the multiple eligible outcome measurements and were graded as having some concerns. Overall, 7 RCTs were graded as having some concerns, and one RCT was graded as high risk.

### 3.2. Patients' Information

A total of 546 poststroke aphasia patients and 96 healthy volunteers were included. The patients' basic information are listed in Table 2. 10 studies focused on the patients for the stroke onset within 6 months, another 4 studies [52–55] reported patients' stroke duration of more than 6 months, and 2 studies did not report the duration of stroke [56, 57]. The duration of aphasia was from 0 days to 2 years. For the type of stroke, 9 studies focused on patients with ischemic stroke, 2 studies reported both ischemic stroke and hemorrhage stroke, and 4 studies did not describe or limit the stroke type. 14 studies reported patients who were right-handed, and 2 studies did not report the handedness [58, 59]. For the type of aphasia, 6 studies reported Broca aphasia, and another 9 studies did not limit the type of aphasia.

### 3.3. Intervention

**Figure 3:**
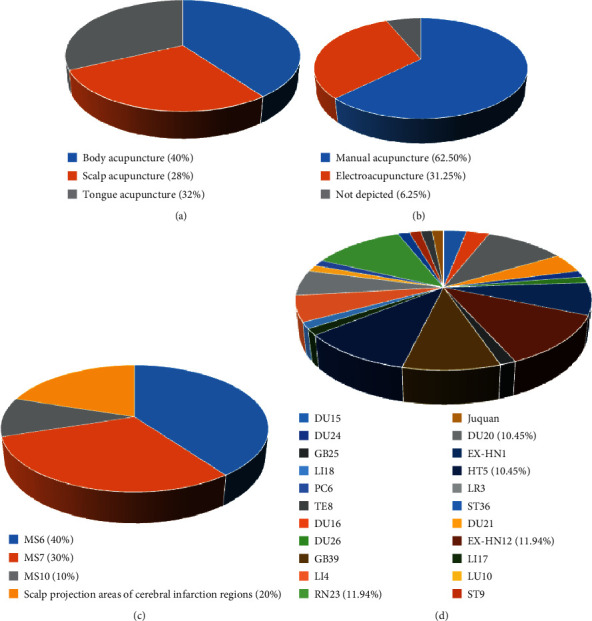
Acupuncture details. Note: (a) acupuncture therapy type: with 40% body acupuncture, 28% scalp acupuncture, and 32% tongue acupuncture. (b) Needling type: with 62.50% manual acupuncture, 31.25% electroacupuncture, and 6.25% not depicted. (c) Scalp acupuncture type: with 40% MS6, 30% MS7, 10% MS10, and 20% scalp projection areas of cerebral infarction regions. (d) Acupoint selection: the acupoints that are used above 10% included RN23 (11.94%), EX-HN12 (11.94%), HT5 (10.45%), and DU20 (10.45%).

According to the STRICTA guideline [66], the needle stimulation included manual acupuncture (10 studies) and electroacupuncture (5 studies). The type of acupuncture therapy included body acupuncture (10 studies), scalp acupuncture (7 studies), and tongue acupuncture (8 studies). For the acupoint selection, all of the 16 studies selected acupoints based on the Traditional Chinese Medicine theory and meridian system. 22 acupoints were mentioned for a total of 64 times in the 16 studies, with RN23 (Lianquan, 8/16) and EX-HN12 (Jinjin and Yuye, 8/16) being the most frequently used acupoints. Other top 4 used acupoints included HT5 (Tongli, 7/16), DU20 (Baihui, 7/16), GB39 (Xuanzhong, 6/16), and EX-HN1 (Sishencong, 5/16). 4 scalp acupuncture areas were involved in 4 studies for 10 times involving MS6 (anterior oblique line of vertex-temporal, the line joining anterior EX-HN1 and GB6 Xuanli), MS7 (posterior oblique line of vertex-temporal, the line joining DU20 and GB7 Qubin), MS10 (anterior temporal line, the line joining GB4 Hanyan and GB6), and the scalp projection areas of cerebral infarction regions [67]. The responses that acupuncture elicited were described as “de qi” in 9 studies. The acupuncture type, needling type, scalp acupuncture areas, and acupoint selection are demonstrated in Figure 3 and Table 3.

### 3.4. Comparison

The included 16 studies contain the following 7 comparison types: acupuncture plus language rehabilitation vs. language rehabilitation [42, 57–59, 64, 65], acupuncture plus language rehabilitation vs. nonpoint needle plus language rehabilitation [45], acupuncture plus language rehabilitation vs. healthy volunteer [56, 62, 63], acupuncture plus conventional treatment vs. conventional treatment [61], acupuncture vs. nonpoint needle [53], acupuncture vs. acupuncture [54, 55], and acupuncture vs. healthy volunteer [60] (Table 3).

### 3.5. Outcome

#### 3.5.1. Aphasia Assessment

**Figure 4:**
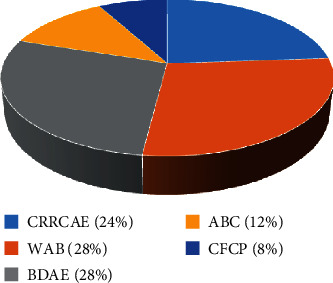
The proportion of aphasia assessment scales. Note: 24% Chinese Rehabilitation Research Center Standard Aphasia Examination (CRRCAE), 28% Western Aphasia Battery (WAB), 28% Boston Diagnostic Aphasia Exam (BDAE), 12% Aphasia Battery of Chinese (ABC), and 8% Chinese Functional Communication Profile (CFCP).

For the poststroke aphasia evaluation, 5 aphasia assessment scales were used including Western Aphasia Battery (WAB; 7 studies; 24.00%), Boston Diagnostic Aphasia Exam (BDAE; 7 studies; 28.00%), Chinese Rehabilitation Research Center Standard Aphasia Examination (CRRCAE; 6 studies; 28.00%), Aphasia Battery of Chinese (ABC; 3 studies; 12.00%), and Chinese Functional Communication Profile (CFCP; 2 studies; 8.00%). The National Institutes of Health Stroke Scale (NIHSS) was used in 2 studies to evaluate neurological function. Meanwhile, activities of daily living (ADL), Stroke-Aphasia Quality of Life-39 (SAQOL-39), and Medical Outcome Study Short Form-36 (SF-36) were applied to assess the activity capability.  Figure 4 shows the proportion of aphasia assessment scales.

#### 3.5.2. fMRI Scanning Method

7 studies conducted task-based fMRI using BOLD to observe the brain activation, with linguistic tasks such as word generation; the other 9 studies conducted rsfMRI to detect the spontaneous brain activity (6 with BOLD technology [52, 53, 56, 59, 60, 62], 2 with DTI technology [45, 63], and 1 with both BOLD and DTI technologies [42]). For the observing time point, 2 studies compared the instant effects of acupuncture [53, 54]; the other 14 studies compared the acupuncture effects after the constant treatments (12 to 30 sessions). The outcome details are listed in Table 4.

#### 3.5.3. Cerebral Response of Constant Acupuncture

*(1) Activation in Broca's Area*. For the studies focused on the effect of constant acupuncture therapy, by observing the signal power of neural activities, studies that used tongue acupuncture and scalp acupuncture reported enhanced activation in Broca's area on poststroke Broca's aphasia patients, compared with participants who received language rehabilitation or conventional treatment [61, 65]; another study with the intervention of scalp acupuncture (MS6 and MS7) found more activated voxels in the projection area of scalp acupuncture, which was located in Broca's area as well as was associated with language function [57]. Additionally, compared with the task of word generation stimulation, a more powerful activation was found in Broca's area after conducting electroacupuncture stimulation on poststroke Broca aphasia patients [54].

*(2) Activation in Mirror-Image Areas of Broca's Area*. For the activation in mirror-image areas, one study reported significant increases of activation areas in the right hemisphere compared to the left hemisphere in the right-hand patients [64]. Another study reported a negative activation in the mirror-image areas on poststroke Broca aphasia patients after receiving tongue acupuncture for one month [61].

As for studies based on ALFF technology, one study reported the decreased ALFF (left temporal pole) and increased ALFF around mirror-image areas of Broca's area (right supramarginal gyrus, right inferior frontal gyrus, and left angular gyrus) in poststroke aphasia patients compared with the healthy volunteers before treatment [60]. After treatment, increased ALFF was found (left temporal pole) on poststroke aphasia patients.

*(3) Brain Functional Connectivity Based on Dual-Stream Model*. For the functional connectivity within the brain neural activity, the dual-stream model was proposed by Hickok and Poeppel [68, 69] and was used to illustrate the speech and language function in terms of brain cortical anatomy. As a complement of the classical Wernicke-Lichtheim model, it enriched the relationship of speech and language impairment and provided a practical way to help to understand the brain network [70, 71]. Based on the dual-stream model, one trial conducted the region of interest (ROI) analysis to detect brain functional connectivity [60]. After acupuncture, a reduction of the functional connectivity inside the dual-stream network was shown. Meanwhile, the intensified functional connectivity between the left inferior temporal gyrus and the middle frontal gyrus can be seen. For the studies based on the analysis of ReHo, the outcomes were partly consistent [52, 58]. Both of the two reported increased ReHo in the brain areas including the right fusiform gyrus, right inferior frontal gyrus, and left superior frontal gyrus and the decreased ReHo in the left inferior temporal gyrus and the right lingual gyrus. Besides that, compared with the control groups after treatment, an increased ReHo in the left anterior cuneiform lobe was detected in patients that received body acupuncture and Schuell language rehabilitation training; also, a decreased ReHo was found in the right anterior central gyrus [58], while patients that received scalp acupuncture and Schuell language rehabilitation training showed an increased ReHo in the right medial temporal lobe and cerebellum and decreased ReHo in the left caudate nucleus and right precentral gyrus [52].

*(4) Brain Functional Connectivity Based on Interactive Model*. The brain function was in an interactive model with complex integrity; thus, the brain network theory was proposed to explore the brain functional activity in detail [72, 73]. Using ICA and other techniques, the functional changes of different brain network models were uncovered [74]; for example, the default-model network was considered to reflect the spontaneous brain activity on the resting state, playing a key role in revealing the self-generated cognitive function [75], and the frontoparietal network was suggested to be associated with the creation of verbal language [76]; the attention network is defined as modulating the orienting, alerting, and executive work, and so on [77]. The damage of the cerebral anatomy structure integrity resulted in the impairment of language function [78, 79]. Compared with healthy participants, impairment was found in the integrity of white matter in poststroke aphasia patients [63]. After a 12-session acupuncture, improvements were shown in the left superior corona radiata, right posterior corona radiata, left external capsule, left superior longitudinal fasciculus, and left superior fronto-occipital fasciculus. In terms of ICA analysis, one study reported that after a 10-session acupuncture, there were reinforced functional connectivity (left superior frontal gyrus, right postcentral gyrus) and decreased functional connectivity (right supramarginal gyrus) within the right frontoparietal network of poststroke aphasia patients [62]. Additionally, decreased functional connectivity (left anterior central gyrus, left middle frontal gyrus) was found within the anterior default-model network. Another study reported that after receiving a 24-session acupuncture therapy, the intensified functional connectivity was shown in the left hemisphere within the frontoparietal network, default-model network, dorsal attention network, ventral attention network, and sensorimotor network of the poststroke aphasia patients, while no significant changes were found in the right hemisphere [45].

*(5) Correlation between fMRI and Clinical Evaluation*. The aphasia quotient (AQ) of WAB was correlated with the fraction anisotropy (FA) value of the left uncinate fasciculus in the poststroke aphasia patients. After treatment, the improvement of AQ was correlated with the ALFF value change in the left TP [42]. Using the functional connectivity analysis method, one trial revealed the AQ changes had a positive correlation with the intensified connectivity between the left inferior temporal gyrus and the middle frontal gyrus [60]. Another study found that the axial diffusivity value of the left superior longitudinal fasciculus had a negative correlation with AQ [63]. For the naming scores of WAB, a positive correlation was observed with the FA value of the right supramarginal gyrus [42] and the left uncinate fasciculus [62], while negative correlations were found in the mean diffusivity (MD) value and radial diffusivity (RD) value of the left superior longitudinal fasciculus [63]. Additionally, the repetition score was proven to have a positive correlation with the FA value of the right supramarginal gyrus [42]. Moreover, spontaneous speech and auditory comprehension were associated with the FA value of the left uncinate fasciculus [62]. Considering the characteristic of Cantonese, Chau's study used the Cantonese Aphasia Battery (CAB), which is the Cantonese version of WAB to assess the language function impairment. The study found a correlation between AQ of CAB and BOLD signal activation in the lesion of Wernicke's speech area on chronic poststroke aphasia patients.

For the CRRCAE score, one study investigated its relation with fractional anisotropy [45]. After treatment, the poststroke aphasia patients in the acupuncture group showed several positive correlations in the following aspects: the reading score and calculation score with the FA value in the right inferior longitudinal fasciculus, the speech score with the FA value in the left cingulum cingulate, the writing score with the left superior longitudinal fasciculus, and the reading score with the right inferior longitudinal fasciculus. Another trial reported that the auditory comprehension function impairment correlated with the lower ReHo value of the temporal gyrus [52].

#### 3.5.4. Cerebral Response of Instant Acupuncture

For the instant effect of acupuncture therapy, Xiao et al.'s study showed that after instant acupuncture stimulation [59], compared with the control group, more activated brain areas emerged in the primary sensorimotor cortex, temporal lobe, occipital lobe, and basal ganglia area of patients in the acupuncture group. One study reported that compared with the activation on the left side of healthy subjects (right-handed), increased neuron activity signals emerged on the lesion side (4 in the left side and 2 in the right side) of poststroke aphasia patients (superior frontal gyrus and middle frontal gyrus) after needling TE 8 with electroacupuncture in a rest-activation model. Similar to the outcome of Liu et al.'s study [64], there were significant increases of activation areas in the right hemisphere compared to the left hemisphere in the right-hand patients [53]. In terms of the correlation among the instant effect, language function, and daily activity ability, improvements in the reading ability, oral speech, and listening comprehension and in the CFCP were shown, as well as in the SAQOL-39 score.

## 4. Discussion

### 4.1. Innovation of This Systematic Review

This systematic review analyzed the mechanism of acupuncture on poststroke aphasia patients through summarizing current clinical neuroimaging researches. Previously, the systematic review and meta-analyses focused on the clinical effects of acupuncture on poststroke aphasia. Both Tang et al.'s study in 2019 and Zhang et al.'s study in 2021 reported acupuncture's clinical effects on poststroke aphasia [15, 17]. To deeply reveal the mechanism of poststroke aphasia's recovery, neuroimaging research was conducted. The existing systematic reviews that intended to explore the neuroimaging mechanism usually concentrated on the specific language recovery hypothesis such as language-related brain region activation or brain functional connectivity. Using meta-analyses, Zhang et al. in 2021 reported the relationship between the damage of dual-pathway tracts and language function impairment, addressing the neural mechanism of the Dual-Pathway White Matter [43]. Du et al.'s study compared regional activation between poststroke aphasia patients and healthy volunteers, implying the significance of dominant and nondominant language networks [24]. Though these studies provided valuable findings, they did not focus on the specific treatment of poststroke aphasia. With the growing number of neuroimaging researches of acupuncture on poststroke aphasia, the multiple models of imaging technologies and various fMRI test indexes provide direct evidence on revealing the neural signals under different conditions. Compared with other systematic reviews, this study analyzed the neural mechanism of acupuncture on poststroke aphasia by summarizing the multiple models of neuroimaging research. Moreover, our study analyzed the correlation between clinical indexes and neuroimaging outcomes, hoping to provide practical methods for the clinic. The findings of neural mechanisms can be summarized as the activation of language-related brain regions and functional changes of brain connectivity.

### 4.2. Hypothesis of Poststroke Aphasia Recovery Mechanism

Based on the classical theories of poststroke aphasia that derived from the anatomical locations, the impairments of Broca's area (the left inferior frontal gyrus) and Wernicke's area (the left superior temporal gyrus) are the most studied hypotheses [80]. Through BOLD-fMRI, significant brain activation induced by acupuncture stimulation was discovered in 14 studies, indicating that acupuncture might be a powerful stimulation for poststroke aphasia patients. By observing the ALFF changes, Zhang et al.'s study showed a correlation between the left temporal pole and AQ after acupuncture therapy, stressing the importance of the left uncinate fasciculus and left temporal pole in the recovery of poststroke aphasia [42]. To evaluate the effect in detail, Li and Yang's study compared acupuncture stimulation with word generation task, and the outcome showed a more powerful efficacy in the acupuncture group [54]. While word generation was a basic process during speech and comprehension rehabilitation, their findings indicated that acupuncture intensified the recovery process and played a role as a complementary therapy. Zhang et al.'s study and Li and Yang's study were consistent with current evidence, for both language-related brain areas and the survived brain structures around language regions, playing roles in the recovery of poststroke aphasia [81]. In accordance, clinical studies applied acupuncture in the projection of the language-related brain areas, proven to have effects in improving repetition and naming functions. Previously, meta-analysis based on clinical trials suggested that the stimulation therapy over language-related brain regions was beneficial for the naming performance [82].

As for findings related to functional changes of brain connectivity, through efforts of language researchers, studies have revealed that the ventral stream accounts for the language comprehension function [83] and the dorsal stream accounts for speech generation and speech perception [68]. Among the included 16 trials, only one study reported the recovery of language function emphasized on the hypothesis of the dual-stream model. According to Zhang's study in 2019 [60], there was a stronger connection within the dual-stream model and a weaker connection between the left inferior temporal gyrus and the right middle frontal gyrus in poststroke aphasia patients compared to the healthy subjects. After treatment, the abnormal connections in poststroke aphasia patients showed a fallback tendency, and the connection intensity between the left inferior temporal gyrus and the right middle frontal gyrus correlated with AQ value and spontaneous speech. The outcomes were consistent with the hypotheses of Fridriksson's study [71], implying that the intensified connection between the dual-stream model in the bilateral hemisphere might be a potential target in poststroke aphasia's recovery. According to recent studies, evidence has been shown that enhanced cerebral blood flow and increased activation of the right hemisphere emerged after stroke, which highlighted the indispensable role of the right hemisphere in the recovery process of aphasia [84, 85].

Beneath the brain function changes, adequate cerebral blood flow perfusion provides the metabolism supports for the neural activities [27]. Researches focused on the cerebral blood of poststroke aphasia patients found worse perfusion in regions around the core lesions [86]. Hypoperfusion in the perilesional tissue correlates with the severity of language impairment. Thus, cerebral perfusion was considered as a prognosis factor that influences the language function recovery process [87, 88]. Substantial evidence was reported of acupuncture's effect on the cerebral perfusion and angiogenesis promotion in the cerebral ischemic condition [89–91], indicating that the boom of collateral circulation which plays as a compensatory part might be a potent pathophysiologic basis. Nevertheless, sparse studies provide limited evidence for acupuncture's effect on the cerebral perfusion in poststroke aphasia patients. Hence, further study could take this area into account.

### 4.3. Current Methodology of Included Studies

#### 4.3.1. Sample Size

According to a literature review of 1461 fMRI studies, the medium sample size of the highly cited studies was 14.5 [92]. The small sample size (Mumford's study, *n* = 30) decreased the reliability of expected effect sizes [93]. Meanwhile, the statistical significance and true effects vary greatly as the sample size changes. Desmond and Glover's study showed that 12 subjects can meet the requirement of the typical activation on a voxel-based level (80% power, *α* = 0.05) [94]. In this study, the medium sample size of the included studies was 27. 8 studies had a sample size below 30 participants, and 8 studies had a sample size between 30 and 100 participants. Considering that the calculation of fMRI is different from other trials, setting a standardized protocol is a practicable way to improve the statistical power, especially in multicenter studies [95].

#### 4.3.2. Blinding and Concealment

Among the 16 included trials, only one study conducted the blinding procedure [45]. Though the therapeutic effects of acupuncture have been widely demonstrated by plenty of studies, the placebo effect was considered in some objective-evaluated studies [96, 97]. To reveal the real effect of acupuncture, the blinding procedure is strongly recommended. Currently, blinding interventions such as blunt needles without penetration, superficial needling, and nonaffected meridian needling are applied in researches [98, 99]. Apart from the acupuncture method, efforts are made to minimize the placebo effect, including limiting interactions between therapists and participants, adding to objective outcome measurements [100]. In this review, fMRI as an objective outcome is less affected by the placebo effect. However, the fMRI is easily affected by even a subtle stimulation, which might cause mixed bias to the specific effect of acupoint-based acupuncture therapy. Thus, the nonmeridian point needling or the nontherapeutic acupoint needling can be applied as a blinding control [101].

#### 4.3.3. Intervention

Among the included 16 studies, one study did not mention the detail of acupuncture, 10 studies performed manual acupuncture, and 5 studies performed electroacupuncture. 9 studies described the acupuncture response as “de qi,” which was induced by manipulation of the practitioner. But none of the “de qi” responses was quantitatively evaluated. In Traditional Chinese Medicine theory, “de qi” response is the core role to ensure the therapeutic effect of acupuncture, making it the ultimate goal in the manipulation of acupuncture practitioners. However, the “de qi” response is perceived mostly by patients and partly felt by the practitioner. Moreover, fMRI recorded the BOLD signal changes according to different “de qi” responses, and the signal activation in the right anterior insula correlated with the “de qi” degree. Thus, the assessment scales help with the visualization of the “de qi” response, providing an objective standard for clinical practice. Currently, there are several assessment scales for acupuncture senses, such as Visual Analog Scales (VAS) that quantified the five acupuncture senses and the anxiety degree and the Massachusetts General Hospital Acupuncture Sensation Scale (MASS) that contains VAS and two subscales which are used to evaluate the acupuncture sensation spreading and patients' mood. Apart from these scales, the Southampton Needling Sensation Questionnaire (SNSQ), Park Questionnaire, and Subjective Acupuncture Sensation Scale (SASS) are frequently used among studies.

#### 4.3.4. Outcome Index

Among the 16 included studies, the most frequently used language assessments were CRRCAE, WAB, BDAE, ABC, and CFCP. WAB and BDAE were the most commonly used assessment tools for aphasia, and both of them were utilized for the clinical diagnosis of neurological disorders. Compared with BDAE, WAB was more popular for the brief design, which provides a quicker and more convenient assessment for clinicians and patients [102]. The WAB was recommended by the Research Outcome Measurement in Aphasia consensus statement to evaluate the poststroke aphasia recovery and is prevalently practiced in western countries [103]. It can be used to diagnose the aphasia type and to measure the aphasia impairment by calculating AQ. The Chinese version of WAB was practicable for the poststroke aphasia assessment for its comprehensive characteristic. However, it remains to be explored whether the accuracy of WAB is influenced by the language diversity and culture gap. Hence, the aphasia assessment tools based on Mandarin were explored. ABC was formulated based on WAB with unified guidelines, scoring standards, pictures and text cards, and aphasia classification according to standardized requirements [104–106]. It can be applied in aphasia patients with different handedness and education level. Moreover, it was sensitive to mild language impairment, and the quantitative result was a practical tool for the clinician. Another tool was CRRCAE, which was designed by the China Rehabilitation Research Center according to the Mandarin characteristic and Chinese culture [107]. It contains the evaluation of general conditions and language function [108]. It was estimated that 91% of aphasia patients could complete the evaluation in items of oral comprehension and listening comprehension, making it prevalent among Chinese aphasia patients [109]. The result of CRRCAE was classified into 6 grades and could be presented as a curve at different phases of aphasia, which provided a visualized way for the aphasia recovery process [110]. Nevertheless, CRRCAE was only applied for adult patients, and the 30 items cost too much time for evaluation. Hence, it requires clinicians to choose the preferred tools to evaluate the aphasia condition correctly.

### 4.4. Limitation

There are still some limitations in this review. Firstly, multiple imaging technologies and fMRI test indexes (ReHo\ALFF\ICA) were conducted in the current included studies. On the one hand, the abundant findings helped to reveal the neural signals under different conditions; on the other hand, the inconsistent study protocols made it hard to carry out the quantitative meta-analyses. Since rigorous meta-analysis is an indispensable tool for providing evidence for healthcare policy and clinical practice [111, 112], future researches should be more cautious about the study design. Secondly, the included studies were all conducted in China, and the included participants were Chinese speakers. Considering the language diversity between Chinese characteristics and English letters, language and publication bias might exist. Thirdly, though most of the studies contained more than 20 participants (14/16), some studies contained less than 10 participants. Given the instability raised from small sample sizes, this might be a potential origin of heterogeneity. Despite 12 subjects being proven to meet the requirement of neuroimaging studies [96], trials with large sample sizes are required to minimize the risks of bias such as inadequate randomization or unstable outcomes. Thus, future studies were needed to deeply explore neuroimaging mechanisms of acupuncture's effects, as well as to provide more evidence to validate current findings.

## 5. Conclusion

In this study, we summarized current evidence of neuroimaging in the effects of acupuncture on poststroke aphasia. Through the systematic review method, we found that the mechanism of acupuncture's effect might be associated with the activation and functional connectivity of language-related brain areas. Moreover, the relationship between specific language function and clinical language function scales was revealed. However, these studies were still in the preliminary stage. Thus, multicenter RCT is needed to verify current evidence. Meanwhile, further neuroimaging mechanisms of acupuncture's effects should be explored to help predict the recovery process of poststroke aphasia.

## Figures and Tables

**Table 1:** Searching strategy and searching terms.

1	Aphasia
2	Acupuncture
3	Electroacupuncture
4	Needle
5	Stroke
6	Apoplexy
7	Cerebral vascular accident
8	Cerebral infarction
9	Magnetic resonance imaging
10	fMRI
11	Diffusion tensor imaging
12	DTI
13	Blood oxygen level dependent
14	BOLD
15	Amplitude of low-frequency fluctuation
16	ALFF
17	Region of interest
18	ROI
19	Regional homogeneity
20	ReHo
21	Independent component analysis
22	ICA
23	Functional connectivity
24	FC
25	Neuroimaging
26	Arterial spin labeling
27	ASL
28	Acupuncture-related terms: 2 OR 3 OR 4
29	Stroke-related terms: 5 OR 6 OR 7 OR 8
30	Neuroimaging-related terms: 9 OR 10 OR 11 OR 12 OR 13 OR 14 OR 15 OR 16 OR 17 OR 18 OR 19 OR 20 OR 21 OR 22 OR 23 OR 24 OR 25 OR 26 OR 27
31	Final searching terms: 1 AND 28 AND 29 AND 30

**Table 2:** The overview of the 16 included studies.

Publication year	First author	Funding organization	fMRI examination time	Study type	Stroke type	Aphasia duration	Type of AG patient	Number of acupuncture group	Type of CG patient	Number of control group	Treatment duration	Handedness	Outcome index
2021	Binlong Zhang [42]	Dongzhimen Hospital Affiliated to Beijing University of Chinese Medicine	Before and after treatment	Observational	Ischemic	1-6 months	Poststroke Broca aphasia	36	Healthy volunteer	24	Not depicted	Right handed	WAB, BDAE
2021	Defu Zhao [58]	Natural Science of Guizhou Province	Before and after treatment	RCT	Not depicted	16 days-5 months	Poststroke aphasia	48	Poststroke aphasia	48	1 month	Not depicted	CRRCAE, MoCA, clinic efficacy
2021	Xiaolin Li [45]	National Research Projects for Public Welfare Industries	Before and after treatment	RCT	Ischemic	14 days-6 months	Poststroke Broca aphasia	21	Poststroke Broca aphasia	20	8 weeks	Right handed	CRRCAE
2019	Binlong Zhang [60]	Dongzhimen Hospital Affiliated to Beijing University of Chinese Medicine	Before and after treatment	Observational	Ischemic	1-6 months	Poststroke aphasia	31	Healthy volunteer	26	1 month	Right handed	WAB, BDAE
2019	Yukang Xiao [59]	Affiliated Hospital of Hubei University of Medicine	Before and after treatment	RCT	Not depicted	0-6 weeks	Poststroke aphasia	50	Poststroke aphasia	50	30 days	Not depicted	CRRCAE, CFCP, SAQOL-39
2018	Mingge Yang [52]	Rehabilitation Hospital Affiliated to Fujian University of Traditional Chinese Medicine	Before and after treatment	RCT	Not limited	Within 2 years	Poststroke Broca aphasia	15	Poststroke Broca aphasia	15	4 weeks	Right handed	CRRCAE, BDAE, SF-36
2018	Shun Li [61]	Guangdong Provincial Science and technology Project	Before and after treatment	RCT	Ischemic	14 days-3 months	Poststroke Broca aphasia	11	Poststroke Broca aphasia	12	1 month	Right handed	CRRCAE, CFCP, ADL
2017	Jingling Chang [53]	Doctoral Fund of the Ministry of Education of China	Instant	Observational	Ischemic or HE	14 days-2 years	Poststroke Broca aphasia	22	Poststroke Broca aphasia	21	Instant acupuncture	Right handed	CRRCAE, BDAE
2016	Aiqin Wang [62]	Dongzhimen Hospital Affiliated to Beijing University of Chinese Medicine	Before and after treatment	Observational	Ischemic in cortical	1-6 months	Poststroke Broca aphasia	10	Healthy volunteer	10	30 days	Right handed	WAB, BDAE
2016	Binlong Zhang [56]	Dongzhimen Hospital Affiliated to Beijing University of Chinese Medicine	Before and after treatment	Observational	11 ischemic and 1 hemorrhage in basal ganglia	—	Poststroke aphasia	12	Healthy volunteer	12	30 days	Right handed	WAB, BDAE
2016	Jinying Liu [63]	Dongzhimen Hospital Affiliated to Beijing University of Chinese Medicine	Before and after treatment	Observational	IS	32-90 days	Poststroke Broca aphasia	10	Healthy volunteer	10	30 days	Right handed	BDAE, WAB
2016	Yuping Yuan [57]	Xinjiang Medical University	Before and after treatment	RCT	Ischemic in the left hemisphere	—	Poststroke aphasia	20	Poststroke aphasia	20	4 weeks	Right handed	ABU, NIHSS
2013	Jinhuan Liu [64]	Research Project of Hubei Provincial Department of Education	Before and after treatment	RCT	Not limited	7-10 days	Poststroke aphasia	10	Poststroke aphasia	10	30 days	Right handed	ABC, clinic efficacy
2012	Ni Li [65]	Affiliated Hospital of Hubei University of Traditional Chinese Medicine	Before and after treatment	RCT	Ischemic	0-6 months	Poststroke Broca aphasia	20	Poststroke Broca aphasia	20	4 weeks	Right handed	ABC, NIHSS, ADL
2011	Geng Li [54]	Hong Kong Jockey Club Charities Trust	Instant	Observational	6 ischemic and 1 hemorrhage in the left hemisphere	More than 6 months	Poststroke aphasia	7	Healthy volunteer	14	Instant acupuncture	Right handed	WAB
2010	Anson C.M. Chau [55]	The study was approved by the institutional review board of the University of Hong Kong/Hospital Authority Hong Kong West Cluster	Before and after treatment	Observational	Ischemic in the left hemisphere	17 ± 8 months	Poststroke aphasia	5	Poststroke aphasia	2	24 days	Right handed	CAB

ABC: Aphasia Battery of Chinese; ABU: Aphasia Battery of Uighur; ADL: activities of daily living; AG: acupuncture group; BDAE: Boston Diagnostic Aphasia Exam; CAB: Cantonese Aphasia Battery; CFCP: Chinese Functional Communication Profile; CG: control group; CRRCAE: China Rehabilitation Research Center Aphasia Examination; MoCA: Montreal Cognitive Assessment; NIHSS: National Institutes of Health Stroke Scale; RCT: randomized controlled trial; SAQOL-39: Stroke-Aphasia Quality of Life-39; SF-36: Medical Outcome Study Short Form-36; WAB: Western Aphasia Battery.

**Table 3:** Intervention details.

Publication year	First author	AG interventions	Needle session	Needle duration	Needle frequency	Needle type	Acupoint	Needle response	CG interventions
2021	Binlong Zhang	Acupuncture and language rehabilitation	Not depicted	Not depicted	Not depicted	Not depicted	Not depicted	Not depicted	Language rehabilitation
2021	Defu Zhao	MA and language rehabilitation	24	30 days	6 times per week	Body acupuncture	DU26 (Shuigou), DU20 (Baihui), DU15 (Yamen), DU16 (Fengfu), DU24 (Shenting)	De qi	Language rehabilitation
2021	Xiaolin Li	MA and language rehabilitation	24	8 weeks	3 times per week	Body acupuncture, scalp acupuncture, tongue acupuncture	HT5 (Tongli), GB39 (Xuanzhong), EX-HN12 (Jinjin, Yuye), RN23 (Lianquan), DU20 (Baihui), EX-HN1 (Sishencong), scalp projection areas of cerebral infarction regions	De qi	Nonpoint needle and language rehabilitation
2019	Binlong Zhang	MA	12	30 days	3 times per week	Body acupuncture, scalp acupuncture, tongue acupuncture	HT5 (Tongli), GB39 (Xuanzhong), EX-HN12 (Jinjin, Yuye), RN23 (Lianquan), DU20 (Baihui), EX-HN1 (Sishencong), scalp projection areas of cerebral infarction regions	De qi	Healthy volunteer
2019	Yukang Xiao	MA and language rehabilitation	30	30 days	Once per day	Scalp acupuncture, tongue acupuncture	MS6, GB25 (Fengchi), DU16 (Fengfu), DU15 (Yamen), RN23 (Lianquan), EX-HN12 (Jinjin, Yuye), ST9 (Renying), LI17 (Tianding), LI18 (Futu), LU10 (Yuji)	Not depicted	Language rehabilitation
2018	Mingge Yang	MA and language rehabilitation	20	4 weeks	5 times per week	Scalp acupuncture	MS6, MS10	De qi	Language rehabilitation
2018	Shun Li	MA and conventional treatment	24	30 days	6 times per week	Body acupuncture	RN23 (Lianquan)	De qi	Conventional treatment
2017	Jingling Chang	EA (2 Hz, 2 mA)	Instant	—	—	Body acupuncture	HT5 (Tongli), GB39 (Xuanzhong)	Not depicted	Nonpoint needle
2016	Aiqin Wang	MA and language rehabilitation	12	30 days	3 times per week	Body acupuncture, tongue acupuncture	HT5 (Tongli), GB39 (Xuanzhong), EX-HN12 (Jinjin, Yuye), RN23 (Lianquan), DU20 (Baihui), EX-HN1 (Sishencong), LI4 (Hegu), LR3 (Taichong)	De qi	Healthy volunteer
2016	Binlong Zhang	MA and language rehabilitation	12	30 days	3 times per week	Body acupuncture, tongue acupuncture	HT5 (Tongli), GB39 (Xuanzhong), EX-HN12 (Jinjin, Yuye), RN23 (Lianquan), DU20 (Baihui), EX-HN1 (Sishencong), LI4 (Hegu), LR3 (Taichong)	Not depicted	Healthy volunteer
2016	Jinying Liu	MA and language rehabilitation	12	30 days	3 times per week	Body acupuncture, tongue acupuncture	EX-HN12 (Jinjin, Yuye), RN23 (Lianquan), DU20 (Baihui), EX-HN1 (Sishencong), LI4 (Hegu), LR3 (Taichong), HT5 (Tongli), GB39 (Xuanzhong)	Not depicted	Healthy volunteer
2016	Yuping Yuan	EA and language rehabilitation	24	4 weeks	6 times per week	Scalp acupuncture	MS6, MS7, DU20 (Baihui)	De qi	Language rehabilitation
2013	Jinhuan Liu	EA and language rehabilitation	12	30 days	3 times per week	Scalp acupuncture, tongue acupuncture	MS7, HT5 (Tongli), DU21 (Qianding), RN23 (Lianquan), EX-HN12 (Jinjin, Yuye)	Not depicted	Language rehabilitation
2012	Ni li	MA and language rehabilitation	20	4 weeks	5 times per week	Scalp acupuncture, tongue acupuncture	MS7, DU21 (Qianding), EX-HN12 (Jinjin, Yuye), Juquan	Not depicted	Language rehabilitation
2011	Geng Li	EA (2 Hz, mild stimulation)	Instant	—	—	Body acupuncture	TE 8 (Sanyangluo)	De qi	EA (2 Hz, mild stimulation)
2010	Anson C.M. Chau	EA	24	8 weeks	3 times per week	Body acupuncture	LI4 (Hegu), PC6 (Neiguan), LR3 (Taichong), ST36 (Zusanli)	De qi	EA

AG: acupuncture group; CG: control group; EA: electroacupuncture; MA: manual acupuncture.

**Table 4:** Neuroimaging outcomes.

Publication year	First author	Sample size	Neuroimaging technologies	Scanning design	Image acquisition time	Comparison	Neuroimaging results
2021	Binlong Zhang	60 (36 poststroke aphasia, 24 healthy volunteers)	rsfMRI, BOLD (ALFF), and DTI (FA)	—	Before and after acupuncture treatment	AG after treatment vs. healthy volunteer in ALFF	Decreased ALFF: the left temporal pole and increased ALFF in the right supramarginal gyrus, right inferior frontal gyrus, and left angular gyrus
							Correlation: the repetition scores have a positive correlation with the FA value of the right supramarginal gyrus
						AG after treatment vs. healthy volunteer in DTI	Decreased FA value: bilateral uncinate fasciculus. No significant difference was found in FA between poststroke aphasia patients before and after treatment.
							Correlation: the amount of damage in the left uncinate fasciculus was associated with WAB-AQ.
2021	Defu Zhao	96 (poststroke aphasia)	rsfMRI, BOLD (ReHo); task-state fMRI, BOLD (task-induced brain activation)	Task state: 20 s; resting state: 20 s; word generation task	Before and after acupuncture treatment	AG after treatment vs. CG after treatment	Increased ReHo: the right fusiform gyrus, right inferior frontal gyrus, left anterior cuneiform lobe, and left superior frontal gyrus. Decreased ReHo: the left inferior temporal gyrus, right lingual gyrus, and right anterior central gyrus.
2021	Xiaolin Li	50 (poststroke aphasia)	rsfMRI, DTI (FA)	—	Before and after acupuncture treatment	AG after treatment vs. AG before treatment	Increases intensified functional connectivity: the left hemisphere within the frontoparietal network, default-model network, dorsal attention network, ventral attention network, and sensorimotor network; no significant changes were found in the right hemisphere.
						AG after treatment vs. CG after treatment	Increased value: brain network connectivity, brain network node efficiency, and local brain network node efficiency in dorsal attention network 2 and frontoparietal network 1; brain network node degree centrality in sensorimotor network 2, ventral attention network 1, and ventral attention network 2.
							Correlation between FA and CRRCAE: FA value in the right inferior longitudinal fasciculus with the reading score and calculation score, FA value in the left cingulate gyrus with the speech score, right inferior longitudinal fasciculus with the reading score, and left superior longitudinal fasciculus with the writing score.
2019	Binlong Zhang	57 (31 poststroke aphasia, 26 healthy volunteers)	rsfMRI, BOLD (ROI)	—	Before and after acupuncture treatment	AG before treatment vs. healthy volunteer	Increased: the connectivity inside the dual-stream network, the connectivity between the dual-stream network and other brain areas except for the opposite dual-stream network. Decreased: the global efficiency of the dual-stream network, the average path length of the left middle gyrus, and correlated with the score of spontaneous speech and BDAE.
						AG after treatment vs. AG before treatment	Increased: the connectivity with other regions (left inferior temporal gyrus to the right middle frontal gyrus). Decreased: the connectivity inside the dual-stream network (the left posterior middle temporal gyrus to the left middle temporal gyrus; left upper middle temporal gyrus to the left middle temporal gyrus).
							Correlation: the ALFF change of the left temporal pole was positively correlated with WAB-AQ change.
2019	Yukang Xiao	100 (poststroke aphasia)	rsfMRI, BOLD (ReHo)	—	Before and after acupuncture treatment	AG before treatment vs. CG before treatment	Activated brain areas: primary sensorimotor cortex (*n* = 6^∗^/8^#^), temporal lobe (*n* = 12^∗^/11^#^), occipital lobe (*n* = 10^∗^/10^#^), and basal ganglia (*n* = 3^∗^/4^#^), but no significant change was found (*P* > 0.05).
						AG after treatment vs. AG before treatment	Activated brain areas: primary sensorimotor cortex (*n* = 35/8), temporal lobe (*n* = 32/11), occipital lobe (*n* = 34/10), and basal ganglia (*n* = 15/4); significant changes were found in the activated areas (*P* < 0.05).
						AG after treatment vs. CG after treatment	Activated brain areas: primary sensorimotor cortex (*n* = 17^∗^/35^#^), temporal lobe (*n* = 15^∗^/32^#^), occipital lobe (*n* = 16^∗^/34^#^), and basal ganglia (*n* = 5^∗^/15^#^); activated areas in AG significantly increased compared with CG (*P* < 0.05).
2018	Mingge Yang	30 (poststroke aphasia)	rsfMRI, BOLD (ROI)	—	Before and after acupuncture treatment	AG after treatment vs. CG after treatment	Increased ReHo: left dorsolateral superior frontal gyrus, insula, precuneus, and calcarine. Right triangle inferior frontal gyrus, fusiform gyrus, medial temporal lobe, and cerebellum. Decreased ReHo: left inferior temporal gyrus, caudate nucleus, right lingual gyrus, and precentral gyrus. Correlation: the auditory comprehension function impairment correlated with the lower ReHo value of the temporal gyrus.
2018	Shun Li	23 (poststroke aphasia)	Task-based fMRI, BOLD (task-induced brain activation)	Task state: 30 s; resting state: 30 s; word generation task	Before and after acupuncture treatment	AG after treatment vs. AG before treatment	Increased activated areas: left hemisphere Broca's area. Negative activated areas: mirror area of Broca's area in the right hemisphere.
						CG after treatment vs. CG before treatment	No significant changes were found in the activated areas; the Broca's area in the left hemisphere and the mirror area of Broca's area in the right hemisphere were symmetrically activated.
2017	Jingling Chang	22 poststroke aphasia	rsfMRI, BOLD (ROI)	Needling state: 30 s; resting state: 30 s	Instant acupuncture	AG after treatment vs. AG before treatment	Increased activated areas: the left hemisphere.
2016	Aiqin Wang	20 (10 poststroke aphasia, 10 healthy volunteers)	rsfMRI, BOLD (ROI)	—	Before and after acupuncture treatment	AG before treatment vs. healthy volunteer	Decreased FC: left frontal parietal network (left inferior frontal gyrus, left inferior parietal lobule); right frontal parietal network (inferior parietal lobule); salience network (the right middle frontal gyrus, right anterior cingulate); anterior default mode network (left angular gyrus); posterior default mode network (right superior frontal gyrus, left superior parietal lobule, right anterior cuneiform lobe, right inferior frontal gyrus).
						AG after treatment vs. AG before treatment	Increased FC: right frontal parietal network (left superior frontal gyrus, right postcentral gyrus). Decreased FC: right frontal parietal network (right supramarginal gyrus); anterior default mode network (left anterior central gyrus, the left middle frontal gyrus). No significant changes of FC were found in the left frontal parietal network, posterior default mode network, and salience network.
							Correlation: the average FC value of decreased brain area in the anterior default mode network (left anterior central gyrus, the left middle frontal gyrus) had a negative correlation with the spontaneous score. The FC of the right frontal parietal network (right supramarginal gyrus) had a positive correlation with AQ, repetition, and naming scores.
2016	Binlong Zhang	24 (12 poststroke aphasia, 12 healthy volunteers)	rsfMRI, BOLD (ICA)	—	Before and after acupuncture treatment	AG before treatment vs. CG before treatment	Increased brain areas: auditory network (right anterior central gyrus). Decreased brain areas: right frontoparietal network, frontoparietal network 1, auditory network (left inferior frontal gyrus, the left middle frontal gyrus, the right middle frontal gyrus).
						AG after treatment vs. AG before treatment	Increased brain areas: executive control network, auditory network (left paracentral lobule, the right middle frontal gyrus, right superior frontal gyrus).
2016	Jinying Liu	10 poststroke aphasia	rsfMRI, DTI (ROI)	—	Before and after acupuncture treatment	AG before treatment vs. healthy volunteer	Impairment of white matter: bilateral external capsule, bilateral uncinate fasciculus, left cingulate gyrus, left anterior limb of the internal capsule, left superior fronto-occipital fasciculus, and left inferior fronto-occipital fasciculus.
						AG after treatment vs. AG before treatment	Increased values: left superior corona radiata (AD), right posterior corona radiata (AD, MD), left external capsule (MD, RD), left superior longitudinal fasciculus (AD, MD, RD), and left superior fronto-occipital fasciculus (AD, MD, RD).
							Correlation: for the left superior longitudinal fasciculus, both of the MD value and RD value had negative correlations with WAB naming scores; the AD value had a negative correlation with AQ.
2016	Yuping Yuan	40 (poststroke aphasia)	Task-based fMRI, BOLD (task-induced brain activation)	Task state: 32 s; resting state: 32 s; word generation task	Before and after acupuncture treatment	AG after treatment vs. AG before treatment	Significant differences were found in activated voxel numbers in brain areas related to language function (*P* < 0.05).
						AG after treatment vs.CG after treatment	No significant changes were found in the poststroke aphasia patients (*P* > 0.05).
2013	Jinhuan Liu	20 (poststroke aphasia)	Task-based fMRI, BOLD (task-induced brain activation)	Task state: 20 s; resting state: 20 s; word generation task	Before and after acupuncture treatment	CG after treatment vs. CG before treatment	Significant changes were found in the following brain areas: bilateral medial frontal gyrus, bilateral middle frontal gyrus, bilateral inferior frontal gyrus, bilateral anterior central gyrus, left angular gyrus, and left posterior superior temporal gyrus.
						AG after treatment vs. AG before treatment	Significant changes were found in the following brain areas: bilateral medial frontal gyrus, bilateral superior frontal gyrus, bilateral middle frontal gyrus, bilateral inferior frontal gyrus, bilateral anterior cuneiform lobe, posterior cingulate cortex, left angular gyrus, left posterior superior temporal gyrus, bilateral cuneus, bilateral lingual gyrus, bilateral inferior occipital gyrus, bilateral basal ganglia, splenium of corpus callosum, and right posterior cerebellar lobe.
						AG after treatment vs. CG after treatment	Significant changes were found in the following brain areas: left superior frontal gyrus, left middle frontal gyrus, bilateral inferior frontal gyrus, left anterior central gyrus, left postcentral gyrus, left paracentral lobule, left posterior superior temporal gyrus, posterior cingulate cortex, bilateral anterior cuneiform lobe, bilateral cuneus, left angular gyrus, bilateral lingual gyrus, right hippocampus, right parahippocampal gyrus, bilateral superior occipital gyrus, bilateral inferior occipital gyrus, right posterior cerebellar lobe, left superior cerebellar lobule, and splenium of corpus callosum.
2012	Ni li	40 (poststroke aphasia)	Task-based fMRI, BOLD (task-induced brain activation)	Task state: 20 s; resting state: 20 s	Before and after acupuncture treatment	AG after treatment vs. AG before treatment	Increased activated areas: Broca's area.
2011	Geng Li	21 (7 poststroke aphasia, 14 healthy volunteers)	Task-based fMRI, BOLD (task-induced brain activation)	Needling state: 45 s; resting state: 45 s; word generation task	Instant acupuncture	AG after acupuncture vs. AG before acupuncture	Significant activation in the left inferior frontal gyrus (opercular part, triangular part or insula), right inferior frontal gyrus, or parietal lobe (Rolandic operculum or triangular part). Strong activation on the lesion side of superior and middle frontal gyrus.
						Healthy volunteer after acupuncture vs. healthy volunteer before acupuncture	Activated brain areas: left superior and middle frontal gyrus, but relatively weaker compared with AG.
						AG after WG vs. AG before WG	Activated brain areas: left inferior frontal gyrus (1 poststroke aphasia) and right inferior frontal gyrus (2 poststroke aphasia).
						AG after acupuncture vs. AG after WG	Activated brain areas: the right insula, left precentral gyrus, right median cingulate, and paracingulate gyrus of the limbic lobe.
							Significantly smaller activation: both sides of superior and middle frontal gyrus induced by acupuncture compared with WG task.
2010	Anson C.M. Chau	7 (poststroke aphasia)	Task-based fMRI, BOLD (task-induced brain activation)	Linguistic task	Before and after acupuncture treatment	Well-recovered group vs. poor-recovered group, both received electroacupuncture	Activated brain areas: the left, middle, and superior temporal gyrus.

^#^AG: acupuncture group; ^∗^CG: control group; AD: axial diffusivity; ALFF: amplitude of low-frequency fluctuation; AQ: aphasia quotient; BOLD: blood oxygen level dependent; CRRCAE: Chinese Rehabilitation Research Center Standard Aphasia Examination; DTI: diffusion tensor imaging; FA: fraction anisotropy; FC: functional connectivity; ICA: independent component analysis; MD: mean diffusivity; RD: radial diffusivity; ReHo: regional homogeneity; ROI: region of interest; rsfMRI: resting-state functional magnetic resonance; WAB: Western Aphasia Battery; WG: word generation.

## Data Availability

The data used to support the findings of this study are from the published literature.

## Supplementary Materials

Supplementary Materials PRISMA 2020 Checklist. It contains the 27 checklist items that pertain to the content of a systematic review, including the title, abstract, introduction, methods, results, discussion, and other information.
